# Lipid metabolism drives podocyte injury in diabetic kidney disease

**DOI:** 10.3389/fimmu.2026.1764023

**Published:** 2026-07-06

**Authors:** Jiazhen Shang, Zhitao Zeng, Shouyu Chai, Xincong Lv, Xiaotian Han, Baoze Ma, Yifan Xu, Rong Wang, Zhimei Lv

**Affiliations:** Department of Nephrology, Shandong Provincial Hospital Affiliated to Shandong First Medical University, Jinan, Shandong, China

**Keywords:** diabetic kidney disease, inflammation, lipid metabolism, podocyte injury, renal fibrosis

## Abstract

Diabetic kidney disease (DKD) is the main cause of end-stage renal disease (ESRD) worldwide. Podocytes are important components of the glomerular filtration barrier, which is located between glomerular capillary endothelial cells and the basement membrane, and is essential for maintaining normal renal function. Its injury is a key factor in the progression of DKD. Lipid homeostasis plays an important role in maintaining the normal physiological function of cells. In recent years, many studies have revealed that lipid metabolism disorders play a core role in driving podocyte injury, but its specific mechanism network has not been systematically characterized. This article systematically reviews the latest evidence from recent years, indicating that lipid metabolism disorders mainly lead to podocyte structure and dysfunction, apoptosis and extracellular matrix deposition through key pathways such as lipid peroxidation, abnormal sphingolipid metabolism and abnormal cholesterol accumulation. In addition, the review summarizes the core regulatory roles of related signaling pathways such as Sterol Regulatory Element-Binding Protein 1 (SREBP1), Peroxisome Proliferator-Activated Receptor α (PPARα) and NOD-like receptor family pyrin domain containing 3 (NLRP3) inflammasome in this process. This review emphasizes that lipid metabolism disorder is a key driver of podocyte injury in DKD. This provides a theoretical basis for a new therapy for DKD that focuses on lipid metabolism as the core strategy. Future research should focus on elucidating the interaction network between these pathways and promoting the clinical transformation of related intervention strategies to delay disease progression.

## 1 Introduction

DKD is a common severe microvascular complication of diabetes and is a major cause of ESRD worldwide (1). The typical pathological features of DKD include persistent proteinuria, a decreased glomerular filtration rate and progressive renal fibrosis (2). Epidemiological studies have shown that among patients with diabetic, approximately 30-40% will eventually develop DKD, which will impose a heavy medical burden (3). Although previous strategies to control blood glucose, blood pressure and block the renin-angiotensin-aldosterone system have delayed the progression of the disease to a certain extent, many patients still inevitably go to renal failure (4–6). This challenging situation urgently requires us to further elucidate the pathogenesis of DKD to identify new intervention targets.

In the complex pathological network of DKD, glomerular podocyte injury is recognized as the central link in the promotion of proteinuria and glomerulosclerosis (7, 8). Podocytes are terminally differentiated epithelial cells that cover the glomerular basement membrane (GBM). Their unique foot processes are intertwined to form a slit diaphragm, which constitutes the last extremely precise structural and functional barrier of glomerular filtration (9). Once podocyte dysfunction, apoptosis or detachment from the basement membrane occurs, irreversible damage to the filtration barrier, a large number of protein leakage, and ultimately lead to glomerular sclerosis (10, 11). In recent years, increasing evidence has shown that podocytes are particularly sensitive to changes in lipid metabolism, and that abnormal lipid metabolism can directly drive podocyte injury (12). Lipid metabolism disorder is also an important risk factor in the pathogenesis of DKD (13). In a state of hyperglycemia, if glucose metabolism is impaired, the homeostasis of lipid metabolism in the kidney is destroyed, resulting in the accumulation of harmful metabolites in the glomerulus. These harmful products include ceramide and oxidized lipids, which destroy the structure and function of podocytes, induce podocyte damage, and subsequently cause a series of kidney damages such as proteinuria and renal fibrosis (14).

This article reviews the mechanism through which changes in lipid metabolism drive podocyte injury in the diabetic environment and key treatment strategies targeting lipid metabolism, to provide new ideas for the precise prevention and treatment of DKD.

**Figure 1:**
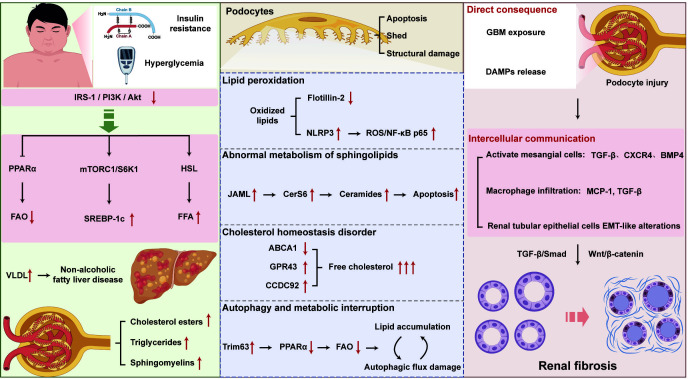
Mechanisms of podocyte injury and renal fibrosis driven by lipid metabolism in diabetic kidney disease. This illustration summarizes the key pathways through which lipid metabolism disruptions contribute to podocyte injury and renal fibrosis in DKD. The process involves three main stages: (1) Systemic Lipid Dysregulation: Insulin resistance promotes hepatic lipid accumulation and increased circulating lipids via SREBP-1c activation, PPARα suppression, and HSL activation. (2) Podocyte Lipotoxicity: Lipid accumulation within podocytes drives injury through oxidative stress, ceramide buildup, cholesterol homeostasis disruption, and impaired FAO/autophagy. (3) Fibrosis Progression: Podocyte detachment and DAMPs release trigger inflammation, mesangial activation, tubular EMT, and ECM deposition, leading to renal fibrosis.

In order to more intuitively summarize the core mechanism of lipid metabolism disorder driving podocyte injury in DKD, this paper constructs the following concept map (**Figure 1**). The following will focus on several key pathways and processes shown in this figure.

## 2 Lipid metabolism in the renal physiological state

### 2.1 The spatiotemporal variability of lipid metabolism within renal glomeruli and tubular structures

Different anatomical regions of the kidney show significant differences in lipid metabolism. Notably, in the glomerular region, podocytes have distinct sphingolipid metabolism conditions. Lipid molecules are the basic components of cell membranes and play crucial roles in signal transduction pathways (15). Unlike those in the glomerular region, renal tubular epithelial cells rely mainly on fatty acid oxidation (FAO) to meet their own energy requirements (16, 17). This metabolic difference reflects all the unique functional requirements of the different renal compartments themselves. Studies conducted using a unilateral nephrectomy mouse model have shown that the glomerular area of the remaining kidney is more susceptible to lipid droplet accumulation caused by a high-fat diet, whereas the renal tubular area is associated mainly with a compensatory increase in mitochondrial oxidation capacity (13, 18). This difference in time and space indicates that different renal regions have varying degrees of vulnerability and adaptive responses to lipid metabolism interruption. Recent evidence further suggests that fatty acid oxidation pathways exhibit cell-type-specific heterogeneity beyond the classical PPARα–long-chain axis, such as distinct medium-chain fatty acid metabolism in tubular epithelial cells (19).

### 2.2 The regulatory network governing lipid homeostasis in podocytes

The homeostasis of podocyte lipids is strictly controlled by a complex and multilevel regulatory network. The discovery of the SREBP family by Brown, Goldstein, and colleagues in the 1990s established the fundamental paradigm that cellular lipid synthesis is transcriptionally regulated by membrane-bound transcription factors activated by sterol depletion (20). Within podocytes, the SIRT1–SREBP1 signaling axis has subsequently been identified as the central pathway controlling cholesterol biosynthesis, and this pathway can regulate the transcriptional activity of SREBP1 (21, 22). In this way, the cholesterol level required for the synthesis of gap junction membrane proteins can be maintained. Sphingolipids are crucial for the structural integrity and signal transduction function of podocytes. Disorders in sphingolipid metabolism can disrupt the integrity of podocytes and accelerate the progression of glomerular diseases by destabilizing cell membranes and promoting inflammatory responses (12). Ceramide synthase 6 (CerS6) expression is elevated in podocytes. This imbalance of CerS6 can promote lipid-induced cytotoxicity by altering sphingolipid metabolism (23). In addition, studies have shown that the expression of junction adhesion molecule-like protein (JAML) is increased in a DKD model. During this process, JAML regulates podocyte lipid metabolism through SIRT1-mediated SREBP1 signaling. In these models, the absence of the JAML function can improve the condition of damaged podocytes and alleviate the problem of proteinuria (24). The expression of proprotein convertase subtilisin/kexin type 9(PCSK9), in podocytes is negatively correlated with intracellular lipid accumulation, which implies that it plays a certain role in maintaining the stable state of these intracellular lipids. Studies have already demonstrated that the upregulation of PCSK9 can exacerbate lipid imbalance, cause mitochondrial dysfunction, and lead to podocyte apoptosis (25, 26).

### 2.3 The physiological roles and protective mechanisms of lipid metabolism in renal function

Healthy kidneys rely on multiple protective mechanisms to regulate lipid metabolism. Within the epithelial cells of renal tubules, the fatty acid oxidation pathway remains active, providing the majority of the energy requirements for the kidneys (15, 17, 27). This metabolic characteristic enables the kidneys to adapt well to the utilization of lipids as substrates. Autophagic flux is crucial for removing damaged organelles and excess lipids. Under conditions of elevated metabolic stress, it plays a key role in maintaining the function of podocytes (28). Research has shown that the overexpression of adipose triglyceride lipase (ATGL) can alleviate oxidative stress and apoptosis by increasing the oxidation capacity of fatty acids and improving mitochondrial function. Such a mechanism has been demonstrated to have a relatively prominent antifibrotic effect in a unilateral ureteral obstruction (UUO) model (29). Furthermore, syndecan-1 has been identified as a regulator of lipid distribution in the kidney. Its expression is negatively correlated with the degree of lipid accumulation, which indicates that maintaining the homeostasis of the extracellular matrix may have a protective effect (30).

## 3 Disorders of lipid metabolism in DKD

### 3.1 Dysregulation of systemic lipid metabolism induced by insulin resistance

Under physiological conditions, insulin mediates its biological effects through binding to the extracellular α subunit of the insulin receptor. This interaction can activate the intrinsic tyrosine kinase activity of the receptor, leading to the recruitment and phosphorylation of insulin receptor substrate-1 (IRS-1). As a result, the phosphatidylinositol 3-kinase (PI3K)/protein kinase B signaling cascade and various downstream pathways are involved in this process, which is helpful for maintaining glucose homeostasis (31). Activated AKT promotes the formation of vesicles containing GLUT4, allowing them to move from the cytoplasm to the plasma membrane, and also facilitating the uptake of glucose by cells (32). Insulin resistance is a core pathological feature of DKD. It disrupts systemic lipid metabolism through various mechanisms. Specifically, insulin overactivates sterol regulatory element-binding protein 1c (SREBP-1c) by taking advantage of the mechanistic target of the rapamycin complex 1 (mTORC1)/S6 kinase 1 pathway. It can also inhibit the oxidation of fatty acids mediated by PPARα. These changes can cause an accumulation of triglycerides in the liver, leading to nonalcoholic fatty liver disease, and can also result in the excessive secretion of very-low-density lipoprotein (VLDL) (33). Under normal circumstances, insulin can inhibit the activity of hormone-sensitive lipase (HSL) through the PI3K/Akt signaling pathway, thereby reducing the release of free fatty acids (FFAs). However, if insulin resistance occurs, HSL activity will increase, which will cause an increase in the amount of FFAs released into the circulation. These increased FFAs are rich substrates for lipid synthesis in the liver. When chronic overnutrition occurs, some metabolic by products, such as hyperglycemia and non-esterified fatty acids, namely NEFAs, are produced. These metabolic by-products can cause damage to the activation of insulin receptors and the downstream IRS-1/PI3K/Akt2 signaling. This damage can lead to chronic inflammation in adipose tissue. It will also cause ectopic lipid deposition in the liver and muscle tissues, and at the same time, it will be accompanied by endoplasmic reticulum stress and oxidative stress (34). These affected organs will interact with each other, and this interaction will make metabolic disorders more severe and also aggravate insulin resistance, thus forming a harmful feedback loop to the body.

### 3.2 The distinctive features of localized lipid accumulation in kidney

In DKD, the kidneys demonstrate a distinct pattern of lipid accumulation. Lipidomic analyses have indicated that lipid deposition within the glomerular region, particularly in podocytes, is highly selective, characterized by the abnormal accumulation of cholesterol esters, triglycerides, and sphingomyelins (24, 27). This lipid accumulation is closely associated with alterations in the expression of local lipid metabolic enzymes in the kidney, such as the specific upregulation of ceramide synthase 6 (CerS6) in podocytes of diabetic murine models (23). At the molecular level, the pathogenesis of lipid accumulation involves dysfunction of the ATP-binding cassette transporter A1 (ABCA1) signaling pathway (35, 36), dysregulation of cholesterol metabolism mediated by G protein-coupled receptor 43 (GPR43) (37), and aberrant activation of the SREBP1 signaling pathway (24, 38, 39). Together, these molecular mechanisms support the selective lipid deposition observed in the kidneys during DKD.

### 3.3 Pathological accumulation of lipid-derived toxic metabolites, including ceramides and oxidized lipids, within the kidney

The harmful effects of certain lipid metabolites are integral to the progression of DKD. Ceramides derived from CerS6 (d18:1/16:0) have been shown to induce mitochondrial oxidative stress and inflammatory responses by interacting with the mitochondrial channel protein VDAC1 (23). At the same time, oxidized lipids exacerbate podocyte injury through mechanisms including promoting cytoskeletal reorganization, inducing insulin resistance, and activating inflammatory pathways (40). Preclinical studies have confirmed that dysregulation of sphingolipid metabolism not only impairs the structural integrity of podocytes but also promotes glomerular disease progression through signal transduction pathways (15, 41, 42). Importantly, these toxic metabolites may establish a deleterious feedback loop, for instance, the accumulation of ceramides can further inhibit SIRT1 activity, thereby exacerbating lipid metabolic disturbances (43, 44).

### 3.4 Epigenetic modulation of lipid metabolism reprogramming

Epigenetic mechanisms play a crucial role in the reprogramming of lipid metabolism associated with DKD. SIRT1-mediated deacetylation regulates lipid homeostasis in podocytes by regulating the SREBP1 signaling pathway (24, 45). Experimental intervention using the DNA methyltransferase inhibitor Adenosine Deaminase (ADA) has demonstrated that the SAHH-NLRP3 inflammasome axis serves as an integrative link between metabolic processes and epigenetic regulation, thereby exacerbating podocyte injury induced by hyperglycemic conditions (46). Furthermore, Trim63 promotes the degradation of PPARα mediated by ubiquitin, which constitutes a novel post transcriptional regulatory mechanism. This mechanism plays a certain role in the damage of fatty acid oxidation and will also cause podocyte damage later (47). These findings elucidate the intricate network of interactions between lipid metabolic dysregulation and epigenetic modifications, providing a molecular framework for understanding the phenomenon of “metabolic memory” in DKD.

## 4 The molecular pathways underlying podocyte damage induced by lipid metabolism

### 4.1 Lipid peroxidation and its impact on the structural integrity of podocyte membranes

In DKD, the hyperglycemic environment induces extensive lipid peroxidation in podocytes, leading to the production of large amounts of oxidized lipid products (48). These oxidized lipids impair the structural integrity of the podocyte membrane, particularly destabilizing lipid raft microdomains (49). Research has indicated that Flotillin-2 (Flot2), a protein associated with lipid rafts, exhibits downregulated expression in DKD models, which exacerbates podocyte injury and proteinuria. Conversely, overexpression of Flot2 has been demonstrated to preserve podocyte morphology and function (50, 51). In addition, lipid peroxidation aggravated podocyte injury by activating NLRP3 inflammasome and enhancing reactive oxygen species (ROS)/NF-κB p65 signaling pathway (52). Critically, NLRP3-derived IL-1β and IL-18 are not freely diffusible but are packaged into exosomes for secretion, a process governed by the ceramide-lysosome-MVB axis. In podocytes, acid sphingomyelinase (Asm)-generated ceramides inhibit lysosome-MVB interaction, diverting MVBs toward exosomal release rather than lysosomal degradation (53). This mechanism, which was originally characterized in D-ribose-induced podocyte stress (53), has been confirmed in metabolic pathologies including obesity-related glomerulopathy, where Asm-ceramide signaling similarly drives NLRP3 activation and inflammatory exosome release (54). Thus, sphingolipid metabolism not only triggers inflammasome activation but also determines how inflammatory signals propagate beyond the podocyte.

### 4.2 Dysregulated sphingomyelin metabolism and the induction of apoptotic pathways in podocytes

Disorders in sphingolipid metabolism are closely related to podocyte injury, which is characterized by the upregulation of CerS6 in the renal podocytes of diabetic mice (23). The aberrant accumulation of ceramides has been shown to induce podocyte apoptosis through mechanisms involving mitochondrial dysfunction and proinflammatory pathways (12, 55–57). Research has indicated that the JAML modulates podocyte sphingolipid metabolism through SIRT1-mediated SREBP1 signaling, and targeted deletion of JAML can improve podocyte injury in diabetic mice (24). In addition, metabolites of sphingomyelin have been demonstrated to affect the stability of the podocyte cytoskeleton altering the lipid microenvironment surrounding diaphragm proteins (23, 58).

### 4.3 Impairment of the occluding membrane protein resulting from cholesterol homeostasis dysregulation

ATP-binding cassette transporter A1 (ABCA1) was first identified in the late 1990s as the molecular defect underlying Tangier disease, and subsequent studies by Oram and Lawn established its central role in mediating cholesterol efflux to lipid-poor apolipoproteins, thereby maintaining cellular cholesterol homeostasis (1). In the context of DKD, disruption of this ABCA1-mediated cholesterol efflux pathway in podocytes represents a key manifestation of cholesterol homeostasis disturbance (36, 59), leading to free cholesterol accumulation and impaired slit diaphragm integrity. G protein-coupled receptor 43 (GPR43),which functions as a post transcriptional regulator of cholesterol metabolism, exacerbates podocyte lipotoxicity upon activation, resulting in abnormal accumulation of free cholesterol within podocytes (37). This disturbance in cholesterol balance directly impairs the proper localization of pore membrane proteins, such as nephrin and podocin in lipid rafts, impairing the filtration barrier function of podocytes (50, 60). Additionally, CCDC92 has been identified as a novel regulator of lipid homeostasis that promotes cholesterol accumulation in podocytes by regulating ABCA1 signaling pathway (35, 61).

### 4.4 Disruption of lipid metabolism and autophagy flux leading to a vicious cycle

In the context of diabetes, impaired autophagic activity and dysregulation of lipid metabolism in podocytes establish a self-sustaining detrimental cycle (62). Trim63 promotes the ubiquitin-mediated degradation of PPARα, leading to defective FAO and subsequent lipid accumulation, which further aggravates podocyte injury and renal fibrosis (47). Experimental evidence suggests that sarsasapogenin can alleviate podocyte injury in diabetic rats by restoring autophagic flux in podocytes (62). In addition, dysfunction of the GADD45α-R-loop pathway disrupts the demethylation process of the STEAP4 promoter, and impairs mitochondrial function and lipid metabolism, thereby increasing oxidative stress in podocytes (63).

## 5 The complex multidimensional pathways underlying podocyte damage that contribute to renal fibrosis

### 5.1 Exposure of the glomerular basement membrane resulting from podocyte detachment

The pathogenesis of renal fibrosis involves a complex interplay between injured parenchymal cells and multiple non-parenchymal cell lineages within the fibrotic niche (64). Recent advances in single-cell and spatial transcriptomics have identified the cellular components and signaling networks driving fibrosis progression, including myofibroblast activation, immune cell infiltration, and extracellular matrix accumulation (64). In DKD, podocyte injury and detachment are critical initiating events that trigger downstream fibrotic cascades, which directly lead to the exposure of the glomerular basement membrane and destruction of its structural integrity (2). Experimental evidence from diabetic mouse models has indicated that targeted deletion of Rho-associated coiled-coil kinase 2 (ROCK2), specifically in fibroblast cells, significantly attenuates proteinuria and glomerular fibrosis, thereby underscoring the key role of fibroblast detachment and subsequent GBM exposure in the onset of fibrosis (65). After exposure to GBM, the transmission of mechanical stress activates mesangial cells, which in turn promotes excessive extracellular matrix deposition and the establishment of a fibrotic microenvironment (66).

### 5.2 The profibrotic role of damage-associated molecular patterns released from injured podocytes

Damage-associated molecular patterns (DAMPs) released from injured podocytes are critically involved in initiating and propagating the inflammatory and fibrotic cascade in the renal environment.

Following podocyte injury, endogenous DAMPs, including mitochondrial DAMPs and extracellular matrix (ECM) components, are released. These molecules are recognized by pattern recognition receptors (PRRs) such as Toll-like receptor 4 (TLR4) and the NLRP3 inflammasome (67–69). In a UUO model, there is an upregulation of both DAMPs and their corresponding receptors, which leads to activation of the NLRP3 inflammasome. This activation contributes to fibrosis, apoptosis, and ROS-mediated damage (69). Similarly, research on systemic sclerosis (SSc) has demonstrated that ECM glycoproteins, including fibronectin containing an EDA domain and tenascin C, function as DAMPs that activate resident stromal cells through TLR4, inducing profibrotic responses and sustained myofibroblast activation (70). DAMPs initiate downstream inflammatory signaling pathways, such as the ERK/EGR1 pathway and the NLRP3 inflammasome, through receptors like TLR4. This signaling cascade promotes the secretion of proinflammatory mediators, which aggravate podocyte injury and renal interstitial fibrosis (37, 67, 70). Mitochondrial DAMPs (mito-DAMPs) have been shown to aggravate liver fibrosis by inducing M1 macrophage polarization in a TREM2-dependent manner, a mechanism that may also be relevant to renal pathology (71). DAMPs released during necroptosis, including various cytokines, can trigger nonsuppurative inflammation, thereby promoting extracellular matrix remodeling and fibrosis (70, 72). Exosomes as lipid-regulated DAMP vectors. Beyond passive DAMP release, podocytes actively secrete exosomes containing IL-1β and IL-18 under lipotoxic stress. This process is controlled by Asm-ceramide-dependent lysosome-MVB trafficking (53). Under diabetic conditions, CerS6-derived ceramides may similarly disrupt lysosome-MVB fusion, promoting exosomal packaging of NLRP3 products. These inflammatory exosomes function as “DAMP vectors” that deliver IL-1β to mesangial cells and macrophages, amplifying glomerular inflammation (54). Notably, this mechanism is metabolism-specific: amitriptyline (Asm inhibitor) or genistein (acid ceramidase activator) blocks both ceramide accumulation and exosomal IL-1β release (53), suggesting that lipid metabolism intervention can simultaneously suppress inflammasome activation and its intercellular dissemination. Moreover, targeted deletion of CLDN5 or WIF1 in podocytes through paracrine mechanisms has been shown to worsen renal fibrosis, whereas systemic administration of WIF1 can mitigate DKD and UUO-induced fibrosis (73). Podocyte injury also contributes to renal tubular damage and interstitial fibrosis through PRKCI-mediated signal transduction, a cross-cellular effect that has been validated in an OASIS transgenic mouse model (74).

### 5.3 Initiation and sustenance of the epithelial-mesenchymal transition of podocytes injury

In DKD, a hyperglycemic environment serves as the primary initiating factor for epithelial-mesenchymal transition (EMT). Elevated glucose levels activate distinct signaling pathways and modulate molecular regulators, inducing phenotypic alterations in podocytes. By acting as the central stimulus, high glucose directly promotes EMT in podocytes, which leads to increased cellular migratory capacity and the development of proteinuria. This phenomenon represents an early stage of renal injury and sets the stage for the subsequent progression of fibrotic changes (75, 76).

Since the early 1990s, TGF-β has been recognized as the prototypical cytokine driving tissue fibrosis across diverse organ systems. The landmark review by Border and Noble in 1994 established TGF-β as the central mediator of extracellular matrix accumulation in kidney disease, providing the foundational framework for subsequent mechanistic studies (12). In DKD, TGF-β1 functions as the principal inducer of EMT in podocytes, facilitating the upregulation of mesenchymal markers such as alpha-smooth muscle actin (α-SMA) and vimentin (77–79). The Wnt/β-catenin signaling pathway is abnormally activated in podocytes and accelerates the fibrotic process by regulating gene expression to reduce epithelial markers, including renin and synaptopodin, while enhancing the expression of mesenchymal markers, such as α-smooth muscle actin (48, 78). Furthermore, the accumulation of ROS triggers activation of the NLRP3 inflammasome and mediates SHP2 signaling, which promotes the interstitial transformation of renal tubular epithelial cells and exacerbates fibrosis (80). Activation of the Yes-associated protein (YAP) and transcriptional enhancer factor 1 (TEF1) pathways also contributes to EMT initiation by upregulating interstitial gene expression and intensifying podocyte injury (77). Studies have confirmed that Sestrin2 overexpression can inhibit TGF-β/Smad and YAP/TEF1 pathways, thereby alleviate the initiation of high-glucose-induced EMT. Conversely, Sestrin2 downregulation exacerbates renal injury in DKD (77). In addition, dysregulation of the long non-coding RNA MALAT1 affects the initiation of EMT in DKD, and its downregulation can promote the transformation of mesenchymal phenotype (81).

EMT sustains irreversible renal fibrosis through a continuous positive feedback mechanism involving epigenetic regulation and inflammatory responses. The TGF-β/Smad and Wnt/β-catenin signaling pathways constitute a positive feedback loop. Specifically, TGF-β1-induced EMT promotes the deposition of ECM components, such as collagen, which in turn further activates TGF-β signaling and ultimately leads to tubular and glomerular sclerosis. At the same time, continuous activation of the Wnt/β-catenin pathway maintains podocytes in a mesenchymal phenotype, enhancing their migratory capacity and ECM synthesis, thus sustaining the fibrotic process. In addition, increased phosphorylation of MAPK pathway components, including ERK, JNK, and p38, also contributes to proinflammatory and profibrotic signaling during EMT, thereby supporting cell phenotypic switching and ECM accumulation (79).

In the context of epigenetic regulation, circPlekha7 modulates the expression of KLF4 and mitofusin2 by targeting miR-493-3p, thereby inhibiting the maintenance of EMT. Downregulation of circPlekha7 or upregulation of miR-493-3p exacerbates the persistence of EMT and promotes fibrosis (82). In addition, studies have shown that long non-coding RNA MALAT1 can alleviate fibrosis in DKD by regulating EMT. But its dysregulation can lead to the persistence of pathological phenotypes (81).Under conditions of oxidative stress, elevated glucose levels induce mitochondrial damage and the accumulation of ROS, which in turn trigger oxidative stress responses and activate EMT-related signaling pathways, such as TGF-β, thereby establishing a deleterious feedback loop. Connexin32 (Cx32) plays a critical role in modulating oxidative stress and EMT processes within renal tubular epithelial cells (83, 84).

Regarding intercellular communication and inflammatory processes, injury to podocytes triggers the dedifferentiation of renal tubular epithelial cells (PTECs) and the manifestation of EMT-like alterations through extracellular vesicles (EVs), thereby contributing to the progression of fibrosis (85). Chronic inflammatory responses, including the activation of the NLRP3 inflammasome and macrophage infiltration, have been shown to enhance the expression of EMT-associated markers, sustain the mesenchymal phenotype, and facilitate ECM accumulation (86).

### 5.4 Crosstalk between podocytes and other cells

Abnormal lipid metabolism not only leads to podocyte injury, but also affects the function of tubular epithelial cells and mesangial cells through intercellular crosstalk (**Table 1**), further accelerating renal fibrosis and renal function decline.

**Table 1:** Summary of lipid metabolism-mediated intercellular crosstalk in renal injury.

Interaction axis	Key molecules/pathways	Cellular effects & pathological processes	Final outcome
Podocyte-Tubular Epithelial Cell	Massive Proteinuria, NF-κB, ROS	Tubular Epithelial Cell Toxic Injury: Protein overload induces lysosomal rupture and oxidative stress.	Tubulointerstitial Inflammation and Fibrosis
		Cellular Phenotypic Transition: Induction of Epithelial-Mesenchymal Transition (EMT) or programmed cell death.	
		Inflammatory Recruitment & Fibrosis Initiation: Secretion of MCP-1, TGF-β1, etc., recruiting macrophages, activating fibroblasts, and leading to ECM deposition.	
Podocyte-Mesangial Cell	TGF-β/Smad, CXCR4, BMP4	Paracrine Signaling Activation: Podocyte-derived factors (e.g., TGF-β, BMP4) directly act on mesangial cells.	Glomerulosclerosis, Mesangial Matrix Expansion
		Mesangial Cell Activation: Promotes mesangial cell proliferation and synthesis of ECM components (e.g., Collagen I, III).	
		Impaired ECM Degradation: Upregulation of TIMPs and inhibition of MMP activity.	
Podocyte-Macrophage-Mesangial Cell Axis	TGF-β, MCP-1, and other inflammatory mediators	Inflammatory Circuit Formation: Podocyte injury releases factors that recruit macrophages to the glomerulus.	Glomerular and Tubulointerstitial Fibrosis
		Signal Amplification: Macrophages further activate mesangial cells via paracrine signaling.	
		Synergistic Pro-fibrotic Effect: Establishes a positive feedback loop, accelerating fibrosis.	

Podocyte injury, as an initial step, leads to the formation of high levels of proteinuria through destruction of the integrity of the glomerular filtration barrier. When these abnormally leaked proteins flow through the renal tubules, they are taken up mainly by epithelial cells in the proximal convoluted tubules (87). When the protein load exceeds the processing capacity of renal tubular epithelial cells, excessive protein accumulation in cells leads to lysosomal overload and rupture, and the released hydrolase directly destroys the integrity of cell membrane, resulting in acute toxic injury of renal tubular epithelial cells (88–90). Abnormal proteins such as immunoglobulin fragments can activate intracellular signaling pathways (91, 92) by binding to pattern recognition receptors on the surface of epithelial cells, among which the activation of NF-κB pathway (93, 94) is the most critical. At the same time, mitochondrial dysfunction leads to a large amount of ROS (94), which aggravates the oxidative stress injury of renal tubular epithelial cells. Under the joint drive of toxic effects and activation of signaling pathways, EMT occurs in renal tubular epithelial cells, and some cells enter an activated state, losing epithelial cell-specific markers such as E-cadherin and expressing mesenchymal cell markers such as α-smooth muscle actin α-SMA, and vimentin (95); another part of the cells underwent programmed cell death due to the activation of the ROS-mediated mitochondrial apoptosis pathway (96). Activated renal tubular epithelial cells also secrete a large number of chemokines and cytokines (95). Among them, monocyte chemoattractant protein-1 (MCP-1) can specifically attract monocytes in peripheral blood to migrate to the renal interstitium and differentiate into macrophages (97). Profibrotic factors such as TGF-β1 directly act on renal interstitial fibroblasts, promote their activation and proliferation, and synthesize ECM such as type I collagen, type III collagen, and fibronectin in large quantities. At the same time, it inhibits the ECM degradation activity of matrix metalloproteinases (MMPs) and up-regulates the expression of tissue inhibitor of metalloproteinase (TIMPs), resulting in excessive deposition of ECM in renal interstitium (95), promote the process of renal fibrosis.

Among signaling pathways, the C-X-C chemokine receptor 4 (CXCR4) pathway is important in mediating the interaction between podocytes and mesangial cells. Experimental data indicate that CXCR4 activation facilitates pathological signaling between these cell types, exacerbating glomerular injury (98). Other than that damage to podocytes triggers activation of the TGF-β signaling pathway, which not only induces podocyte apoptosis but also exerts paracrine effects on mesangial cells, promoting extracellular matrix accumulation and fibrosis (77, 99), and smad3 activation in podocytes plays a critical role in driving mesangial cell proliferation and fibrotic processes (100). Inflammatory responses and immune cell involvement are also integral to this intercellular crosstalk. In the context of DKD, inflammatory mediators released following podocyte injury, such as TGF-β, recruit macrophages that infiltrate the glomerulus. These macrophages, through paracrine signaling, activate mesangial cells, establishing a podocyte-macrophage-mesangial cell inflammatory amplification loop that accelerates renal fibrosis (101). Metabolic dysregulation and oxidative stress further influence this intercellular communication. Activation of the free fatty acid receptor 4 (FFAR4) through its agonist TUG891 ameliorates podocyte injury and reduces renal fibrosis. These findings suggest that metabolic disturbances in podocytes modulate mesangial cell function through paracrine signaling (102). Additionally, overexpression of Sestrin2 inhibited TGF-β/Smad and YAP/TEF1 pathways, and alleviated podocyte injury and mesangial cell proliferation, while reducing extracellular matrix deposition (77). Structural remodeling and apoptosis initiate a series of pathological events. Podocyte apoptosis or shedding exposes the glomerular basement membrane, thereby triggering mesangial cell hyperproliferation and matrix expansion, which are typical features of DKD (103, 104). Single-cell RNA sequencing analyses have confirmed that factors released following podocyte injury, such as bone morphogenetic protein 4 (BMP4), directly regulate mesangial cell phenotypic transformation (105). Moreover, aberrant secretion of vasoactive substances post-podocyte damage disrupts the stability of mesangial-endothelial cell interactions, thereby exacerbating glomerulosclerosis (106, 107).

## 6 Advances in the identification of diagnostic biomarkers and therapeutic approaches

### 6.1 Lipidomics-based screening for early diagnostic biomarkers

In recent years, advancements in targeted lipidomics analysis have introduced novel perspectives for the early diagnosis of DKD. Research has demonstrated that dysregulated lipid metabolism is an independent risk factor for renal injury, which is particularly evident in unilateral nephrectomy mouse models, in which alterations in lipid profiles induced by a high-fat diet markedly influence podocyte structure and function (13). Lipidomic investigations have revealed the accumulation of cholesteryl ester 20:4 in podocytes, a phenomenon associated with mitochondrial dysfunction and impaired autophagy, which may contribute to the progressive deterioration of renal architecture and function. This lipid accumulation is detectable during the early stages of DKD, and is a promising target for early diagnostic interventions (13). Furthermore, metabolomics analyses have identified phosphatidylethanolamine as a critical factor in the initial renal damage observed in DKD, with its aberrant levels serving as a potential biomarker for early detection, particularly in the initial phases of diabetes-related renal pathology (108).

### 6.2 Targeting lipid metabolism pathways

The SREBP1 signaling pathway serves as a central regulatory mechanism that governs lipid metabolism in podocytes. Dysregulation of this pathway results in cellular damage by promoting increased cholesterol uptake and suppressing autophagy. In the context of DKD, the activation of GPR43 upregulates SREBP1 expression through the ERK1/2 signaling cascade, increases cholesterol influx mediated by the LDL receptor (LDLR), and inhibits LC3 maturation along with p62 degradation. These effects collectively exacerbate lipid accumulation, leading to podocyte lipotoxicity and injury. Genetic ablation of GPR43 has been shown to mitigate this pathological process (37). GPR43 acts as a posttranscriptional regulator of cholesterol metabolism and regulates podocyte function through the AMPK signaling pathway. In diabetic mouse models, GPR43 activation promotes Akt phosphorylation, ameliorates insulin resistance, reduces lipid deposition, and ultimately attenuates podocyte injury associated with DKD (31). Furthermore, JAML influences podocyte lipid metabolism through SIRT1-mediated regulation of SREBP1 signaling. SIRT1 activity diminishes SREBP1 function, thereby decreasing lipid synthesis and accumulation. Targeted knockout of JAML significantly improves podocyte damage and proteinuria in diabetic mice (24).

PPARα represents a critical molecular target in podocytes. PPARα contributes primarily to the regulation of lipid metabolic homeostasis by modulating FAO processes. The E3 ubiquitin ligase Trim63 facilitates the ubiquitin-mediated proteasomal degradation of PPARα, which subsequently results in podocyte injury. Pharmacological or genetic inhibition of Trim63 has been shown to restore PPARα activity and enhance fatty acid oxidation functionality, thereby improving podocyte function. The PPARα agonist fenofibrate promotes fatty acid oxidation (47). Although the ability of fenofibrate to reduce albuminuria in patients with diabetes, its renal outcomes remain inconsistent across trials, likely due to insufficient podocyte-specific drug delivery and off-target hepatic effects. Trim63 inhibitors represent a more targeted preclinical strategy that restores fatty acid oxidation without systemic PPARα activation, potentially circumventing these limitations (48).

Activation of the NLRP3 inflammasome has been implicated in lipid accumulation and injury in podocytes. Intervention with the selective inhibitor MCC950, or genetic ablation of NLRP3, has been shown to mitigate glomerular lipid deposition, decrease podocyte depletion, and ameliorate diabetes-induced renal damage (52). However MCC950 remains preclinical; no NLRP3 inhibitor is currently approved for DKD. In addition, targeting NLRP3 alone may be insufficient if ceramide-driven exosome release persists as a parallel inflammatory pathway. Future strategies should consider the dual inhibition of NLRP3 activation and ceramide-mediated exosome biogenesis to block both cytokine generation and intercellular propagation (53).

Recent work has significantly advanced our understanding of podocyte FFAR4 by demonstrating that podocyte-specific FFAR4 deletion aggravates glomerular damage in adriamycin-induced nephropathy, diabetic, and aging mice. Mechanistically, FFAR4 reduction triggered cellular senescence and lipid metabolism disorder, whereas FFAR4 agonism—including not only TUG891 but also fish oil—exertes anti-senescent and anti-lipotoxic effects via CaMKKβ-AMPK signaling. These findings identify fish oil as a clinically accessible therapeutic option (102).

CXCR4, which serves as the receptor for stromal cell-derived factor 1 alpha (SDF-1α) and is expressed on stromal cells, is critically involved in the mediation of podocyte injury caused by oxidative stress. The inhibition of CXCR4 activity has been shown to ameliorate albuminuria and renal fibrosis, thereby representing a promising therapeutic target (109) (**Table 2**).

**Table 2:** Potential therapeutic strategies targeting lipid metabolism.

Therapeutic targets/strategies	Representing drug/intervention methods	Main mechanism	Protective effect on podocyte/kidney	References
SREBP1 Signaling Pathway	Targeting upstream regulatory factors(as SIRT1 agonists)	Activate upstream molecules such as SIRT1, inhibit the transcriptional activity or nuclear translocation of SREBP1, and reduce cholesterol/fatty acid synthesis.	Reduce lipid accumulation and restore autophagic flux	(24, 37)
	JAML gene knockout	Inhibition of SREBP1 signaling pathway through SIRT1	Improve podocyte injury and reduce proteinuria	(24)
PPARα/FAO	Trim63 inhibitor	Prevent the ubiquitination degradation of PPARα and enhance fatty acid oxidation.	Reduce lipid accumulation and improve mitochondrial function	(47)
	fenofibrate	PPARα agonist	Promote fatty acid oxidation	(17)
NLRP3 inflammasome	MCC950	Selective inhibition of NLRP3 activation	Reduce glomerular lipid deposition, podocyte loss and inflammation	(52)
	SAHH inhibitors	Regulating SAHH-NLRP3 signaling axis	Improve podocyte injury and renal fibrosis	(46)
FFAR4 receptor	TUG891	FFAR4 agonists, Activating of CaMMKβ-AMPK pathway	Alleviate cell senescence and lipotoxicity, protect podocytes	(102)
CXCR4 signal axis	CXCR4 antagonist	Blocking CXCR4-mediated pathological signals	Reduce oxidative stress-induced podocyte injury and improve fibrosis	(109)
Others	Saponin	Restoring autophagic flux	Reduce podocyte injury	(62)
	Germacrone	Inhibition of podocyte apoptosis	Improve renal injury	(110)
	NRG4	Activation of Akt-GSK-3β pathway	Inhibition of podocyte apoptosis	(111)
	Gandi capsules	Regulating SIRT1/AMPK/HNF4A pathway	Improve lipid metabolism of podocytes	(113)

However, several controversies remain unresolved. First, whether SREBP1 activation is a primary driver or secondary response to hyperglycemia remains unclear. The temporal relationship between SREBP1 upregulation and podocyte injury requires further investigation. Second, although PPARα agonists such as fenofibrate promote fatty acid oxidation, their clinical efficacy in DKD is inconsistent, possibly due to off-target effects in nonrenal tissues. Third, the positioning of NLRP3 in the pathogenic cascade is debated: whether it is upstream of lipid accumulation or a downstream consequence of mitochondrial dysfunction. These uncertainties complicate therapeutic strategies targeting these pathways.

### 6.3 Research and clinical utilization of podocyte protective agents

Many studies have shown that certain compounds have protective effects on podocytes. Ophiopogonin, a saponin extracted from Ophiopogon, has been shown to mitigate podocyte injury in diabetic rat models by restoring autophagic function (62). Germacrone has been reported to ameliorate renal damage through the inhibition of podocyte apoptosis (110). Neuregulin 4 (NRG4), which originates from brown adipose tissue, suppresses podocyte apoptosis through the Akt-GSK-3β signaling pathway (111). The FFAR4 agonist TUG891 has been shown to reduce podocyte injury and renal fibrosis in DKD models (102, 112). Furthermore, preclinical investigations indicate that Gandi capsules exert therapeutic benefits by modulating the lipid metabolism network in podocytes (113). But natural products such as sarsasapogenin, germacrone, and Gandi capsules suffer from poor bioavailability, a lack of standardized formulations, and the absence of rigorous clinical trial evidence. Their translational potential remains unclear and lacks pharmacokinetic validation.

### 6.4 Optimization of combined strategies for antifibrotic treatment

Combined therapies targeting lipid metabolism and inflammatory processes have shown considerable therapeutic potential. A dual approach aimed at correcting abnormal cholesterol metabolism and modulating the inflammatory response of mesangial cells has been shown to be effective at delaying the progression of renal fibrosis (114). The inhibition of NLRP3 inflammasome activation not only diminishes lipid accumulation in podocytes but also disrupts the profibrotic signaling cascade mediated by IL-1β/ROS/NF-κB p65 (52). S-adenosylhomocysteine hydrolase (SAHH) inhibitors regulate NLRP3 inflammasome activity, and ameliorate podocyte injury and renal fibrosis (46).

### 6.5 Accurate determination of intervention timing grounded in metabolic reprogramming

Research has indicated that the phenomenon of metabolic memory necessitates prompt intervention. In models of DKD, CCDC92 exacerbates podocyte injury by modulating lipid homeostasis through the ABCA1 signaling pathway. Early targeting of this pathway may yield more significant therapeutic benefits (35). Activators of sirtuins have demonstrated protective effects against various glomerular disorders associated with podocyte damage, underscoring the extensive therapeutic potential of metabolic regulation (45). Single-cell sequencing analyses have elucidated the temporal reprogramming of podocyte lipid metabolism in response to high glucose exposure, thereby informing the identification of optimal intervention timeframes (115). Additionally, therapeutic approaches aimed at activating glycolysis require customization based on the specific stage of disease progression (116). However metabolic memory may render late-stage lipid interventions ineffective, because prior hyperglycemia perpetuates podocyte dysfunction despite glycemic control. Validated biomarkers for identifying optimal intervention windows are currently lacking, hindering clinical implementation.

## 7 Summary and core conclusion

### 7.1 The pivotal role of lipid metabolism dysregulation in DKD

Recent research has established that disorders of lipid metabolism serve as a principal driving force in the onset and progression of DKD. Many animal studies have demonstrated that abnormalities in lipid metabolism induced by a high-fat diet markedly exacerbate podocyte injury in mice subjected to unilateral nephrectomy, as evidenced by distinct pathological features including lipid droplet accumulation and vacuolation. At the molecular level, dysregulated sphingolipid metabolism in podocytes, disrupted cholesterol homeostasis, and the accumulation of lipotoxic substances such as ceramides form a complex pathological network under diabetic conditions. Notably, the identification of novel regulatory mechanisms, including the JAML/SIRT1/SREBP1 signaling axis and the CCDC92/ABCA1 pathway, further elucidates the important role of lipid metabolic reprogramming in DKD. These insights offer a novel metabolic framework for understanding the pathogenesis of DKD.

### 7.2 Injury to podocytes represents a critical target for therapeutic intervention

Podocyte injury is recognized as a critical component of the initial pathological alterations in DKD and serves as the primary contributor to proteinuria. Research has demonstrated that the targeted deletion of JAML or CCDC92 in podocytes markedly ameliorates podocyte injury and reduces proteinuria in diabetic mouse models. Conversely, the downregulation of lipid microdomain-associated proteins, such as Flotillin-2, exacerbates podocyte damage. Mechanistically, podocyte injury results from a combination of factors, including membrane structural impairment due to lipid peroxidation, activation of apoptotic pathways induced by dysregulated sphingolipid metabolism, and disruption of cholesterol homeostasis affecting gap junction proteins. Furthermore, the recognition that ceramides regulate NLRP3 inflammasome product release via exosomes (12, 23, 58) establishes a unified lipid-immune framework: sphingolipid metabolism not only directly injures podocytes but also controls the spatial dissemination of inflammatory signals, positioning podocyte lipid reprogramming as both the origin and propagator of DKD pathology. These insights underscore the key role of podocyte preservation as a therapeutic strategy in DKD and provide a foundational framework for the development of targeted interventions.

### 7.3 Optimization of the path from mechanism research to clinical application

Podocyte injury has been identified as a critical component of the early pathological alterations observed in DKD. It is recognized as the primary contributor to proteinuria. Current studies are progressing from elucidating the fundamental mechanisms toward clinical translation, encompassing multitiered intervention strategies. From a diagnostic perspective, the utilization of lipidomics technology offers novel approaches for the identification of early biomarkers. With respect to therapeutic strategies, pharmacological agents targeting pivotal regulators of lipid metabolism, such as SREBPs, PPARs have demonstrated promising clinical potential. Sirtuin activators and other podocyte-protective compounds have shown broad spectrum efficacy in various glomerular pathologies. Notably, conventional lipid-lowering therapies alone may be insufficient to reverse the pathological features of DKD, underscoring the need for precise interventions aimed at correcting localized lipid metabolic disturbances in renal tissue.

Despite significant progress, several critical questions remain unanswered. (1) Causality vs. correlation: While SREBP1, PPARα, and NLRP3 are implicated in podocyte lipotoxicity, whether they represent independent parallel pathways or a hierarchical cascade is unknown. (2) Model limitations: Current evidence relies heavily on STZ-induced or db/db mouse models, which may not fully recapitulate human DKD pathophysiology. Human podocyte-specific lipidomic data are lacking. (3) Therapeutic translation: Although MCC950 shows promise in db/db mice, the clinical application of NLRP3 inhibitors requires long-term safety validation. Similarly, the metabolic memory phenomenon suggests that early intervention may be critical, yet optimal timing and targets remain undefined. (4) Precision medicine: Given the heterogeneity of DKD phenotypes, stratifying patients based on specific lipid metabolic profiles may be necessary for effective targeted therapy.

Future research should prioritize the characterization of organ-specific lipotoxicity and enhance integrative multiomics approaches to refine the timing and selection of therapeutic interventions.
